# CITES and the Zoonotic Disease Content in International Wildlife Trade

**DOI:** 10.1007/s10640-020-00456-7

**Published:** 2020-08-04

**Authors:** Stefan Borsky, Hannah Hennighausen, Andrea Leiter, Keith Williges

**Affiliations:** 1 Wegener Center for Climate and Global Change, University of Graz, Brandhofgasse 5, AT-8010 Graz, Austria; 2 Faculty of Economics and Statistics, University of Innsbruck, Universitaetsstrasse 15, AT-6020 Innsbruck, Austria

**Keywords:** Zoonotic diseases, International wildlife trade, CITES, Gravity model, F18, F53, Q27, Q54

## Abstract

International trade in wildlife is one contributing factor to zoonotic disease risk. Using descriptive statistics, this paper shows that in the last decades, the volume and pattern of internationally traded wildlife has changed considerably and, with it, the zoonotic pathogens that are traded. In an econometric analysis, we give evidence that an international environmental trade agreement could be used to limit the spread of zoonotic pathogens and disease. More specifically, combining zoonotic disease data with wildlife trade data from the Convention on International Trade in Endangered Species of Wildlife and Fauna (CITES), we show that making trade requirements more stringent leads to a decrease in the number of animals traded and, incidentally, also the number of zoonotic diseases that are traded. Our results contribute to the discussion of policy measures that manage the spread of zoonotic diseases.

## Introduction

Infectious diseases have a substantial negative impact on global health and economies (Morens et al. 2004). They account for over 7 million annual deaths worldwide and cause economic losses through mortality, treatment costs and lost productivity (Fonkwo 2008; WHO 2018b).

Zoonotic diseases, defined as diseases that naturally transmit from animals to humans, are a major contributing factor to human infectious disease risk (Cleaveland et al. 2007). Seventy-five percent of all new, emerging infectious diseases are zoonotic, of which the majority have their origins in wildlife (Jones et al. 2008). Recent pandemics of wildlife-origin infectious diseases, like HIV, SARS, and COVID-19, suggest that further research about activities that bring animals and humans in close contact is needed (Wolfe et al. 2007).

The U.S. National Academy of Medicine defines international trade and commerce as one of the six contributing factors to emerging infectious disease risk (Nature 2011). Trade requires close contact between humans, animals and animal products during the extraction and exchange process, elevating the risk of a zoonotic pathogen crossing species lines (Smith et al. 2012). Moreover, once a pathogen has crossed the species line, domestic and international travel networks increase the risk of the infection spreading to susceptible populations (Perrings et al. 2018).

Recent trends in international wildlife trade are making zoonotic diseases in humans more likely, posing a challenge for disease risk management and public health (Chomel 2009). Increased commercialization of wildlife, as well as tighter trade networks, have created a situation in which the scale and speed of zoonotic pathogen movement are historically unprecedented (Karesh et al. 2005; Cleaveland et al. 2007; Chomel 2009). Examples from the past few decades include the import of the Ebola virus in primates, monkeypox in African rodents and possibly HIV in chimpanzees (Guarner et al. 2004; Keele et al. 2008).

This paper has two goals. First, we describe the volume and pattern of potential zoonotic disease inherent in international wildlife trade. Second, in an empirical exercise, we assess the possibility for international agreements to limit the spread of zoonotic pathogens that occurs through wildlife trade. To answer these research questions we use data on wildlife trade from the Convention on International Trade in Endangered Species of Wildlife and Fauna (CITES), combine it with recent research on the zoonotic virus content of terrestrial animal species from Johnson et al. (2020) and empirically examine the trade flows with a gravity model of trade.

The findings from our econometric analysis suggest that an international trade agreement would manage zoonotic disease risk by controlling how often humans and animals come into contact during wildlife trade. Our results show that making trade requirements more stringent leads to a decrease in the number of animals traded and, incidentally, also the number of potential zoonotic diseases that are traded. This paper contributes to descriptive work about the linkages between wildlife trade and zoonotic disease movement (e.g. Pavlin et al. (2009) and Smith et al. (2012)), causal studies assessing the influence of CITES on trade (e.g. Uscamaita and Bodmer (2009) and Doukakis et al. (2012)) and the economics of infectious disease risk (e.g. Horan et al. (2008) and Perrings et al. (2014)).

## Background and Previous Literature

### Zoonotic Disease and Wildlife Trade

Zoonotic diseases are the result of pathogens—such as viruses, bacteria and parasites—that jump from vertebrate animals to humans (Perrings et al. 2018). Once the pathogen is in the first human, it can be spread to other humans, with the probability of outbreak depending on the infectious period length, mortality, host immunity and human behavior (Anderson and May 1991). Of the approximately 1400 pathogens in the human pathogen database, 61% are known to be zoonotic (Cleaveland et al. 2007). Researchers have estimated that there are between 650,000 and 840,000 existing zoonotic pathogens that could cross the species barrier but have not yet (Perrings et al. 2018). Examples of major zoonotic diseases that have their origins in wildlife include Ebola, tuberculosis, bird flu and COVID-19.

Zoonotic diseases are transmitted from animals to humans through behaviors that bring humans into direct or indirect contact with animals and animal products. Rabies, for example, is spread through direct contact in the form of scratching and biting. Influenza is transmitted through contact with a contaminated environment and malaria is transmitted through an intermediate species, the mosquito. Zoonotic diseases can also be spread to humans through indirect contact, such as coming into contact with the animal’s living space, and through the consumption of contaminated food and water (Cleaveland et al. 2007; CDC 2015).

Every day, wild animals, birds and reptiles are captured, sold, pushed through trading centers and delivered to the end consumer. The trading process contains a number of potential contact points including with hunters, middle-marketers, handlers, and buyers (Karesh et al. 2005). Researchers estimate that every year there are over one billion contacts between wildlife and humans or domestic animals from trade (Karesh et al. 2005). For the majority of the traded wildlife, there is no mandatory testing for pathogens (Smith et al. 2009).

One example of wildlife trade and zoonotic disease transmission is the 2003 outbreak of monkeypox in the United States. The disease was introduced to the U.S. through a shipment of infected African rodents. After the rodents were imported to the U.S., they were housed in close proximity to prairie dogs, which were later sold as pets. The monkeypox outbreak comprised of 47 cases across six states, and was the first time that monkeypox in humans was reported outside of Africa (CDC 2018).

The U.S. Centers for Disease Control responded to the monkeypox outbreak by restricting the import of African rodents (CDC 2018). Import regulations of potentially infected species are a common way for countries to deal with zoonotic disease risk from trade (Can et al. 2019). Nearly all government initiatives are reactive, focusing their efforts on containing zoonotic diseases that have already established themselves in the human population (Smith et al. 2009).

Perhaps the most prominent application of a trade ban in response to zoonotic disease risk was the European Union’s 2007 ban on the import of wild-caught birds in an attempt to curb the spread of avian influenza (Can et al. 2019). While the ban was effective in eliminating legal wild bird trade to Europe, it also opened new trade routes that spread the birds and disease risk to new world regions. As a result, researchers have called for internationally coordinated policies, and not only national or regional regulation, to more effectively manage zoonotic disease risk arising from wildlife trade (Reino et al. 2017).

### CITES

The Convention on International Trade in Endangered Species of Wild Fauna and Flora (CITES)^1^ is a multilateral agreement with the aim to regulate international wildlife trade such that it does not threaten species’ survival in the wild. The convention came into force on July 1st 1975. Today, 183 countries have ratified the agreement.

CITES provides varying degrees of protection to approximately 5,800 animal species and 30,000 plant species, which are grouped in three Appendices based on the effects of the species’ trade on their sustainability in the wild. Appendix I includes all species threatened with extinction; trade in such species is permitted only in exceptional circumstances. Appendix II species are not threatened with extinction but may be if trade is not closely controlled. Appendix III species are included by request from countries that already provide them with protection but need other countries’ cooperation in preventing these species’ unsustainable exploitation. Today, around 1,000 species are covered by Appendix I, 34,500 species by Appendix II, and 200 species by Appendix III. Depending on their abundance, a particular species may be assigned to one Appendix in one country and another Appendix in another country. Species may also change Appendices from year-to-year within one country. This might occur when the species’ population changes.

Every trade flow of an animal of a CITES-listed species to or from a signatory country must be documented. Appendix I species require both an export permit from the country of origin and an import permit from the destination country. Appendix II species require only an export permit. For species covered by Appendix III, an export permit is only required for trade from the “requesting” countries. For all other countries, only a certificate of origin is needed.

Compliance with CITES requires that signatory countries enforce national legislation that implements all aspects of the convention. In case of non-compliance, the Conference of the Parties and the Standing Committee recommends suspension of trade in CITES-listed species with the non-compliant country until they return to compliance. Currently, 25 countries are subject to trade suspensions of CITES-listed species.^2^

## Empirical Strategy

The goal of this paper is to explore the potential of an international treaty to shape and limit zoonotic disease trade. We do this by, first, evaluating the efficacy of the CITES Appendix system in limiting animal trade and, second, estimating its impact on zoonotic disease trade. We hypothesize that moving a species from the more lax Appendix II categorization to the more stringent Appendix I categorization causes a decrease in the number of animals of that species that are traded. As CITES’s mission is not concerned with zoonotic diseases and any estimated effects of CITES on zoonotic disease trade would be unintended, we do not have a hypothesis for the latter question.

We model disease and animal trade with the structural gravity equation developed by Anderson and van Wincoop (2003). In formulating our estimation model we follow the recommendations of Silva and Tenreyro (2006) and Yotov et al. (2016). Accordingly, we apply a poisson pseudo maximum likelihood (ppml) model with exporter-year, importer-year, bilateral and species fixed effects. Our empirical specification is:

1 $$\begin{aligned} \begin{aligned} X_{ijst}&=\exp \left( \alpha _1 CITESapp_{ist} + \alpha _2 RTA_{ijt} + \alpha _3 Specimens_{ist} \right. \\&\quad \left. + \xi _{it}+\delta _{jt} + \zeta _{ij} + \psi _s \right) + \epsilon _{ijst} \end{aligned} \end{aligned}$$

The unit of observation is a bilateral species trade flow in a specific year. The dependent variable, $X_{ijst}$, measures either the *number of animals traded*, or the traded *potential zoonotic disease volume* between exporter *i* and importer *j* of species *s* in year *t*.

Our key variable of interest, *CITESapp*$_{ist}$, captures the stringency of the CITES Appendix system. It is an indicator variable equal to one if species *s* in country *i* in year *t* is listed in Appendix I, zero otherwise. A significant coefficient estimate implies that a country’s volume of potential zoonotic disease traded changes when species are moved from an Appendix II listing to an Appendix I listing. The CITES Appendix system can affect the average volume of potential zoonotic disease traded through two channels: (i) by reducing the overall quantity of the species traded and (ii) by changing the species composition of export flows by regulating species differently.

$\xi _{it}$, $\delta _{jt}$, $\zeta _{ij}$ and $\psi _s$ represent exporter-year, importer-year, bilateral and species fixed effects, respectively. The fixed effects control for country-time factors that influence trade volumes, such as the trading partners’ market size, a country’s biodiversity, specific industry structures, e.g., bio-medical industry, or the multilateral resistance terms, which capture the average trade costs of the exporting and importing countries. Bilateral time-invariant distance measures like geographic distance or a common border are accounted for with the bilateral fixed effects. The species fixed effects capture all species-specific characteristics such as species-specific trade costs and the overall abundance of a species.

In addition to a rich set of fixed effects, we include an indicator variable for the existence of a regional trade agreement (*RTA*$_{ijt}$), which serves as a time varying proxy for bilateral transaction costs. The dummy variable *Specimens*$_{ist}$ indicates whether species are traded as “scientific” specimens. The error term, $\epsilon _{ijst}$, is clustered at the country-pair level.

Our identifying variation is the variation in how a single species is categorized in CITES’s Appendix system. The variation may be temporal and within a single country, or between countries, meaning that exporting countries have categorized the same species differently in the same year. Through our empirical set-up, we are able to disentangle the impact of the Appendix system from confounding factors that affect how much a single species is traded.

## The Data

### Data Description and Modification

#### Data on International Trade in CITES-Listed Animals

The CITES Secretariat—through the UNEP World Conservation Monitoring Centre—records trade permit issuance from its member countries. The database contains trade records of over 34,000 plant and animal taxa, documenting importer and exporter permit issuance at a predominately species-level, and specifying the form in which the species is traded (e.g. live, plant cuttings, manufactured produces, or various appendages and derivatives). The database also indicates which CITES Appendix the traded species falls under. As we focus on zoonotic disease transmission from terrestrial animals to humans, we obtained information on trade flows of all CITES-listed species in the class *mamalia*, for the years 1975 to 2019.

Animals could be traded for a number of different reasons. The majority of animals in our dataset were traded for zoo (26.73%) and commercial purposes (21.83%). Animals traded for scientific (13.87%), circus or travelling exhibition (12.80%), and medical (5.00%) purposes also constitute a large proportion of the trade. The remainder of animals were traded for breeding, personal, etc reasons. Due to inconsistencies in trade purpose reporting (Robinson and Sinovas 2018), we collapse our data across all purpose types.

#### Linking CITES Trades with Data on Zoonotic Disease Content

Information on zoonotic disease content in different species comes from Johnson et al. (2020), who, through a review of the literature, catalogued the presence (or lack thereof) of 139 zoonotic viruses in 5,335 wild terrestrial animal species. Their output is a dataset recording the number of zoonotic viruses each species potentially harbors.^3^

We linked the CITES trade data to Johnson et al. (2020)’s data to generate a variable equal to the potential volume of diseases traded with each traded animal. This was done by matching taxon values from the trade data (indicating animal genus and species) to corresponding genus-species taxons from the zoonotic disease data. As the CITES data is, in some cases, vague or incomplete, missing values from the initial matching were estimated in instances where (i) CITES data did not list a species, but merely that a trade occurred with an animal of a given genus, (ii) the trade data listed a hybrid species of a given genus or (iii) the trade data species was not contained in the zoonotic disease data. In these cases, the zoonotic disease content was estimated by applying the genus-wide average virus count for the missing value.

Of the initial trade dataset of 128,679 observations, 88% were matched to species contained in the zoonotic disease dataset. Addressing unknown or missing species and hybrid species resulted in matches for 92% of all observations. The remainder consists mainly of aquatic animals for which no zoonotic disease data is available, erroneous entries or cases where only the order, rather than genus-species, is specified. The CITES dataset covers 25% of all terrestrial land animals from the zoonotic disease dataset, which have been observed to potentially carry at least one virus.

### International Wildlife Trade and Zoonotic Diseases: Descriptive Evidence

The data set linking CITES-recorded trade flows and disease potential contains 114,160 observations. As we analyze international trade volumes for the period 1975-2018, we dropped those observations for which (i) no information on the quantity traded is available, (ii) the quantity is not measured as the number of animals traded, (iii) the trade occurred outside the time frame 1975-2018 and (iv) observations that refer to domestic trade. We also dropped trade observations of dead animals (e.g. meat) and species listed in Appendix III of the CITES agreement.^4^ After these adjustments, our sample amounts to 45,921 observations that we use to present the descriptive evidence.

Across the entire study period, more than half (53.59%) of the volume of potential zoonotic disease traded originated in the United States. The shares of the second and third largest exporters, Germany and Great Britain, are considerably lower at 7.66% and 6.20%, respectively. In total, the 10 largest exporters account for 90.17% of the total disease trade in CITES-listed animals. In terms of imports, Canada and the U.S. dominate and constitute 34.40% and 26.85% of the potential zoonotic disease trade. Italy is the third largest importer with 9.19% of total imports. The 10 largest disease importers account for 91.37% of total zoonotic disease trade. Table 1 summarizes the export and import shares for the largest traders during the study period.

**Table 1:** Trade shares in disease traded of 10 largest traders

Exporter	Share	Importer	Share
United States	53.59	Canada	32.40
Germany	7.66	United States	26.85
Great Britain	6.20	Italy	9.19
China	5.91	Great Britain	5.25
Vietnam	4.72	Switzerland	5.00
Mauritius	2.85	Japan	4.02
Canada	2.45	Germany	2.97
Singapore	2.39	France	2.31
Indonesia	2.36	Australia	1.83
Philippines	2.04	China	1.55

Trade shares refer to the volume of potential zoonotic disease that is traded. For the years 1975 to 2018, total volume of potential trade amounted to 101.47 million

**Fig. 1:**
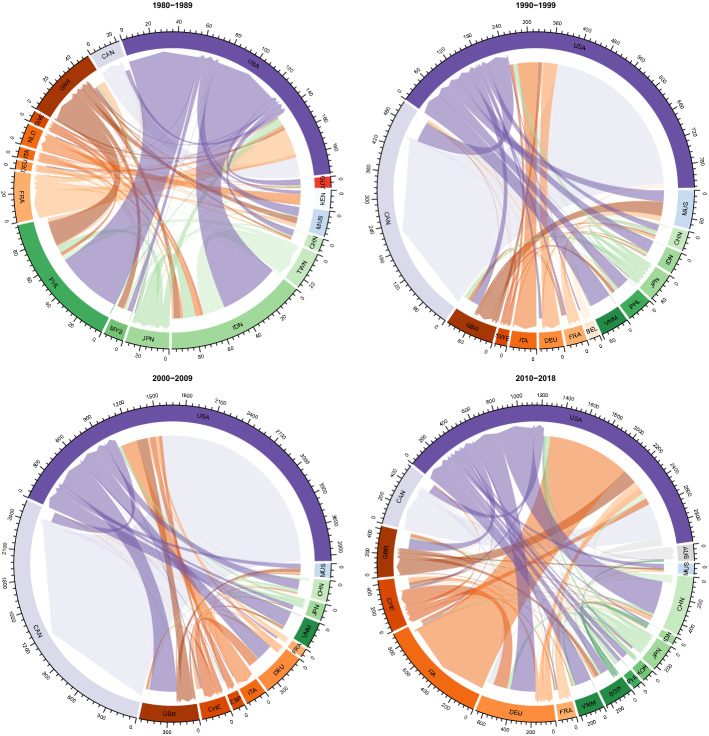
Mean potential zoonotic disease trade flows (in thousands of viruses) for the periods indicated. Flows of at least 1% of the total flows in the given period are depicted. Direction of flow is depicted by a pointed flow end at the importing country, with flow colors corresponding to the destination country. Continents are indicated by differing color groups (North America: purple; Europe: orange; Asia: green; Africa: blue; South America: red; and Oceania: gray). (Color figure online)

Figure 1 illustrates the mean potential zoonotic disease trade for four time periods: 1980–1989, 1990–1999, 2000–2009 and 2010–2018. The trade landscape has shifted over the past four decades. Between 1980 and 1989, the Philippines and Indonesia accounted for the majority of exports. Since then, the title has shifted to the U.S. and Germany, with a smaller but still substantial portion of exports coming from China and other Asian countries. Between 1990 and 2010 the U.S. and Canada accounted for the majority of all trade flows, including both imports and exports. The picture has shifted in the past decade, however, with Italy taking an especially prominent role in imports.

Across the entire study period, the U.S. traded the most number of unique species at 365 types. Germany traded the second most number of unique species (239 species), followed by Canada (201 species), Great Britain (184 species) and France (176 species). On average, the U.S. traded 70 unique species every year. Once we account for the volume of potential zoonotic disease traded (i.e. disease content times quantity of animals traded) we see that either the macaca mulatta (USA) or the macaca fascicularis (Germany, Canada, Great Britain, France) accounts for the largest exporter trade shares.

Table 2 lists, for the four time periods, the CITES-listed species with the highest mean volume of potential zoonotic disease traded. Table 2 shows several trends at once. First, in all four periods, one of the two macaque species, either macaca fascicularis or macaca mulatta, show the highest average volume of potential zoonotic disease traded. Second, the average traded zoonotic disease potential of the two macaque species is significantly higher than for the other species. Third, from the third period (2000–2009) onwards, a significant increase in the volume of potential zoonotic disease traded is observed. Fourth, the ranking of the five most important species in terms of the volume of potential zoonotic disease traded changes over time.

**Table 2:** Disease content of CITES-listed species

Mean volume of disease traded						
Taxon	Order	Virus/unit	1980–1989	1990–1999	2000–2009	2010–2018
*Macaca fascicularis*	Primates	9	5525.83 (1)	6392.92 (2)	20,477.72 (2)	21,447.90 (1)
*Papio ursinus*	Primates	3	883.50 (2)	2593.05 (3)	797.17 (9)	1089.46 (12)
*Macaca mulatta*	Primates	11	681.08 (3)	8203.59 (1)	32,538.17 (1)	10,327.90 (2)
*Clorocebus aethiops*	Primates	6	589.87 (4)	2489.00 (4)	10,321.93 (3)	6391.19 (4)
*Macaca nemestrina*	Primates	6	375.21 (5)	832.85 (5)	78.39 (36)	221.61 (31)
*Papio spp.*	Primates	2.6	40.11 (17)	19.07 (60)	7630.13 (4)	1303.90 (9)
*Chlorocebus sabaeus*	Primates	3	NA (–)	400.85 (10)	1314.36 (5)	8723.65 (3)
*Pteropus alecto*	Chiroptera	6	NA (–)	12.00 (74)	6.00 (119)	4418.21 (5)

Numbers in parentheses denote the rank of the species in that time period with respect to the mean volume of potential zoonotic disease traded

Figure 3 in the appendix graphs the number of CITES-listed species over time and by CITES Appendix categorization. The number of species listed by CITES has largely increased over time—especially in the early years of CITES— likely reflecting changes in species’ abundance in the wild and changes in political will. Except in the initial two years, the number of Appendix II species is always larger than the number of Appendix I species. Appendix I has a minimum of 6 species in 1975 and maximum of 104 species in 1998. For Appendix II, the minimum is 3 species in 1975 and 169 species in 2009.

### Descriptive Statistics

For the econometric analysis, we dropped observations that have insufficient variation after taking into account the exporter-year, importer year, bilateral, and species fixed effects. The sample used to estimate the econometric model contains 32,047 observed trade flows of CITES-listed animals. The sample spans 44 years (1975–2018). There are 148 exporters, 161 importers and 223 species that potentially carry at least one zoonotic disease. Tables 5 and 6 in the “Appendix” define the variables used in the econometric analysis and the variables’ summary statistics.

## Results

Our empirical results are based on the structural gravity model developed in Sect. 3 and were estimated using a ppml model as recommended by Silva and Tenreyro (2006). Columns (1) and (2) in Table 3 present the results using the full sample of CITES-listed species as described in Sect. 4.3. As indicated by the coefficient on $CITESapp_{ist}$ in column (1), a move from Appendix II to Appendix I significantly reduces the quantity of animals traded by 58 %. Column (2) shows that a switch to the stricter Appendix reduces the volume of potential zoonotic disease traded by 72 %.

The difference in the coefficient sizes comes from a heterogenous impact of CITES: species that harbor many zoonotic diseases tend to also respond more strongly to CITES’s Appendix system than species that carry only few zoonotic diseases. To emphasize this point, the second and third parts of Table 3 demonstrate the CITES impact for different subsamples. Columns (3–4) are based on a subsample of species with disease content below the median disease content of 2; columns (5–6) are based on a subsample of species with disease content above the median. Within each subsample, the impact of CITES on animal and disease trade is nearly identical. However, comparing across subsamples, CITES has approximately half the effect in the subsample with disease content less than the median compared to the subsample with disease content more than the median. For example, CITES reduced potential zoonotic disease trade by 53% for the species with few zoonotic diseases. For the species with many zoonotic diseases, CITES reduced disease trade by 83%. The 30 percentage point difference between the two species groups can be explained by the fact that the two groups happen to respond differently to CITES: those with high disease content see a large decrease in the number of animals traded while species with a low disease content see a relatively smaller number of animals traded. This is an unintended effect of CITES.

**Table 3:** CITES and the volume of zoonotic disease trade

	Overall		Below median		Above median	
	(1)	(2)	(3)	(4)	(5)	(6)
$$\hbox {CITESapp}_{{ist}}$$	$$-$$ 0.874$$^{*}$$	$$-$$ 1.277$$^{**}$$	$$-$$ 0.727$$^{**}$$	$$-$$ 0.750$$^{**}$$	$$-$$ 1.742$$^{***}$$	$$-$$ 1.799$$^{***}$$
	(0.475)	(0.526)	(0.334)	(0.355)	(0.546)	(0.541)
$$\hbox {RTA}_{{ijt}}$$	0.794$$^{***}$$	0.820$$^{***}$$	$$-$$ 0.067	$$-$$ 0.169	0.859$$^{***}$$	0.825$$^{***}$$
	(0.260)	(0.277)	(0.206)	(0.237)	(0.288)	(0.285)
$$\hbox {Specimens}_{{ist}}$$	1.874$$^{***}$$	1.859$$^{***}$$	3.105$$^{***}$$	3.087$$^{***}$$	1.831$$^{***}$$	1.851$$^{***}$$
	(0.547)	(0.603)	(0.313)	(0.421)	(0.558)	(0.606)
Importer-year FE	Yes		Yes		Yes	
Exporter-year FE	Yes		Yes		Yes	
Bilateral FE	Yes		Yes		Yes	
Taxon FE	Yes		Yes		Yes	
Observations	32,047		18,750		14,750	
Pseudo R$^{2}$	0.873	0.892	0.933	0.958	0.859	0.868

Dependent Variable in columns (1), (3) and (5): Quantity of animals traded. Dependent variable in columns (2), (4), (6): Volume of potential zoonotic disease traded. Below (above) median refers to a subsample of species, which are below (above) the median in the potential zoonotic disease content. $$^*$$, $$^{**}$$, $$^{***}$$ indicate 10, 5, 1 % significance levels. Standard errors are in parentheses and clustered at the exporter-importer level. Constant included but not reported

### Robustness Checks and Extensions

Although the CITES Appendix regulation is not intended to reduce the volume of potential zoonotic diseases that are traded, its effect on the type of species that are traded is not random. To test for the non-random impact of the CITES Appendix regulation on the volume of potential zoonotic diseases traded, we applied a placebo treatment by creating a random assignment of potential zoonotic disease content to the species in our sample. We estimated specification 1 with the artificially-created volume of potential zoonotic disease. As the disease content now contains no systematic information we expect the coefficient on the CITES Appendix dummy to be similar to the respective coefficient when using the quantity of animals traded as dependent variable. The empirical evidence in column (1) of Table 4 supports this argument. The parameter estimate of the CITES Appendix depicted in column (1) of Table 4 represents roughly the impact on the volume of trade as shown in column (1) of Table 3.

**Table 4:** Robustness checks and extension

	(1)	(2)	(3)	(4)
$$\hbox {CITESapp}_{{ist}}$$	$$-$$ 0.852	$$-$$ 1.208$$^{**}$$	$$-$$ 1.069$$^{**}$$	$$-$$ 1.206$$^{**}$$
	(0.540)	(0.507)	(0.427)	(0.500)
$$\hbox {RTA}_{{ijt}}$$	0.983$$^{***}$$	0.621$$^{*}$$	0.341	0.665$$^{**}$$
	(0.274)	(0.360)	(0.240)	(0.283)
$$\hbox {Specimens}_{{ist}}$$	2.023$$^{***}$$	3.418$$^{***}$$	0.784$$^{**}$$	1.877$$^{***}$$
	(0.546)	(0.224)	(0.347)	(0.631)
$$\hbox {CITESapp}_{{ist}}$$*$$\hbox {Corrup}_{{it}}$$				0.670$$^{***}$$
				(0.167)
Exporter-year FE	Yes	Yes	Yes	Yes
Importer-year FE	Yes	Yes	Yes	Yes
Bilateral FE	Yes	Yes	Yes	Yes
Taxon FE	Yes	Yes	Yes	Yes
Observations	32,047	26,753	27,071	18,027
Pseudo R$^{2}$	0.868	0.964	0.913	0.884

Dependent variable is the volume of potential zoonotic disease traded. $$^*$$, $$^{**}$$, $$^{***}$$ indicate 10, 5, 1 % significance levels. Standard errors in parentheses are clustered at the exporter-importer level. Constant included but not reported. (1) placebo treatment: random disease assignment; (2) subsample excluding the *macaca fascicularis* and the *macaca mulatta*; (3) subsample excluding U.S. as exporting country; (4) interaction with corruption

**Fig. 2:**
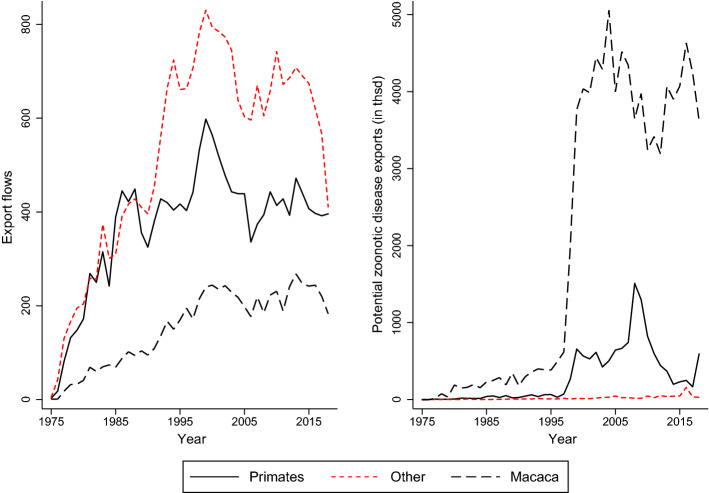
Development of number of exports and volume of zoonotic disease trade over time

**Fig. 3:**
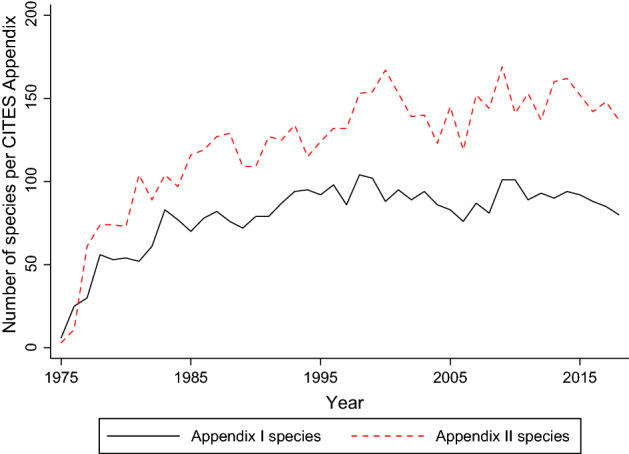
Number of species per CITES Appendix over time

Since 1995, trade in primates has increased considerably. In terms of the number of trade flows, primates were traded as often as all other species together. In terms of volume of potential zoonotic disease traded, primates make up 99% of the total trade volume analyzed in this paper. Figure 2 depicts trade development for primates, all other orders and macacas, which are the major species among primates. The number of export flows are shown in the left panel of Fig. 2. The size of the zoonotic disease trade is shown in the right panel of the same figure. Since the mid-90s, the volume of potential zoonotic disease traded in macacas has increased substantially from increasing demand in the fields of biomedical and bio-warfare research (Eudey 2008).^5^

Within the macacas, two species are traded extensively—the *macaca fascicularis* and *macaca mulatta*. Coincidentally, these two animals lead the CITES-listed animals with regard to their potential zoonotic disease content. To test whether these two animals drive the parameter estimates in our main specification, we estimated specification 1 on a subsample excluding all export flows of the *macaca fascicularis* and *macaca mulatta*. Table 4 column (2) gives evidence that our estimate on the CITES effect is robust to the exclusion of these two animals and the parameter estimate is only slightly smaller compared to our full sample estimates.

With an average 47% of the total volume of potential zoonotic disease traded in CITES-listed animals, the U.S. is by far the largest exporter in our sample. To get a better understanding of how strongly this concentration of trade flows affects our results, we estimate specification 1 on a subsample excluding export flows originating in the United States. The outcome of this robustness exercise is depicted in column (3) from Table 4 and shows that our parameter estimate of the CITES Appendix measure is slightly smaller in size but still statistically significant.

To ensure that CITES functions as it is meant to, member countries must adopt and enforce supporting domestic measures. However, implementation of the CITES regulatory framework and compliance with the CITES Appendix system can be undermined by weak domestic legislation and governance (Reeve 2006).^6^ Non-compliance with CITES regulations results in a larger quantity traded than is recommended by the convention to ensure the survival of the species. While our approach controls for a country’s quality of domestic legislation and governance via exporter- and importer-year fixed effects, it would be interesting to see how institutional quality affects the implementation of CITES regulations and consequently the volume of potential zoonotic diseases traded. In an extension exercise, we account for a country’s institutional quality by introducing a country level measure of perceived corruption stemming from the world governance indicators^7^ and interact it with the CITES Appendix regulation scheme. Column (4) in Table 4 shows the results of this extension exercise. A country’s institutional environment indeed plays a significant role in the implementation of the CITES Appendix system and volume of potential zoonotic disease traded. An increasing level of perceived corruption in an exporting country dampens the decrease in the volume of the potential zoonotic disease traded caused by the CITES Appendix system.

## Policy Discussion and Conclusions

In February 2018, the World Health Organization placed “Disease X” on their shortlist of blueprint priority diseases. “Disease X” was the placeholder name for a hypothetical, unknown pathogen that would cause the next pandemic. The pathogen would likely have zoonotic origins and cross the species line in a place where humans and animals come into close contact (WHO 2018a).

According to experts, COVID-19 is “Disease X”. It is also the illustration of a larger, increasing trend in the risk of pandemics. As humans encroach onto wildlife refugia and wildlife trade brings zoonotic pathogens to cities around the world, disease experts believe that governments should shape their policies to preemptively manage the processes that drive zoonotic disease risk, rather than responding to each individual disease ex-post (Daszak 2020).

This paper gives evidence that an international trade agreement could be effective in managing zoonotic disease risk by limiting the number of contacts between humans and animals. Our results show that CITES’s Appendix system reduces the trade of CITES-listed animals, and with that, inadvertently reduces the volume of potential zoonotic diseases traded. Our results suggest that a hypothetical trade agreement in which there would be trade restrictions on animals with a high risk of passing disease to humans might yield a similarly positive outcome.

At a fundamental level, wildlife trade is an externality problem. The people involved in wildlife trade, like hunters, traders and consumers, impose a cost on society by contributing to zoonotic disease risk. Their private decision-making likely does not account for societal costs, in turn leading to an overabundance of zoonotic disease trade. International coordination like a trade agreement could be used to force the people engaged in wildlife trade to internalize the externality into their private decision-making and move societies closer to the social optimum.

We see this work as timely because of the current momentum brought by the COVID-19 pandemic. In the past, attention and policy responses to zoonotic disease risk from wildlife trade have been limited to the few years after a pandemic. As the outbreak becomes an increasingly distant memory, so does the urgency of the problem (Kennedy and Southern 2020). Zoonotic disease risk, however, does not go away without permanent changes in how humans and animals interact. This paper adds to a growing wave of literature focused on mitigating zoonotic disease risk in the modern era.

There are several limitations to our study and findings. First, our CITES data does not capture the true dynamics of trade flows as we do not have information about illegal wildlife trade. Should illegal trade substitute for legal trade, then our estimated impact of the CITES Appendix system on all international animal and zoonotic disease trade would be upward biased. We also ignore domestic wildlife trade which, as is evidenced by the COVID-19 pandemic, is an important pathway for zoonotic disease movement. Finally, we do not observe the real disease transmission and outbreak risk of trade. That would require knowing, for example, pathogen loads in the traded animals, transmission risks at different contact points and within-country potentials to spread.

That said, we see three promising channels for further research. First, connecting the potential zoonotic disease trade presented in this paper with measures for how easily each pathogen crosses species lines and spreads in communities would allow for closer estimation of the real pandemic risk of wildlife trade. This could be done by collecting information about pathogen loads within animals, protective measures in place at contact points, as well as modeling human behavior in the face of disease risk. Second, accounting for illegal trade would reduce bias in our estimates and provide understanding of the substitutability of illegal for legal wildlife trade in the face of trade agreements. Lastly, analyzing domestic trade of zoonotic viruses would give a fuller picture of zoonotic pathogen movement.

## Appendix

See Tables 5 and 6 and Fig. 3.

**Table 5:** Variable description and sources

Variable	Description	Source
Dependent variables		
$X_{ijst}$	Volume of potential zoonotic disease traded in species *s* between exporter *i* to importer *j* in year *t*.	Johnson et al. (2020) and CITES
Independent variables		
*CITESapp*$_{ist}$	Dummy variable = 1 if the species is listed in the Appendix I of the CITES agreement; 0 if the species is listed in Appendix II.	UNEP-WCMC (Comps.) (2020)
*Specimens*$_{ist}$	Dummy variable = 1 if the species traded is classified as “scientific specimens”; 0 if the species is not a scientific specimen.	UNEP-WCMC and CITES
*RTA*$_{ijt}$	Dummy variable = 1 if a regional trade agreement between the two trading partners is in force in year *t*, 0 otherwise.	Egger and Larch (2008)
*Corruption*$_{it}$	Indicator variable, which captures perceptions of the extent to which public power is exercised for private gain, including both petty and grand forms of corruption, as well as “capture” of the state by elites and private interests with higher values indicating higher level of perceived corruption.	Word Governance Indicators

**Table 6:** Summary statistics

	Observations	Mean	St.dev	Min	Max
Dependent variable					
Disease export (units)$_{ijst}$	32,047	3,143.96	39,276.95	0.07	2,127,763
Quantity (units)$_{ijst}$	32,047	376.97	4,222.40	0.20	193,433
Independent variables					
$\hbox {Virus}_{{s}}$	32,047	3.75	3.10	0.67	11
$\hbox {CITESapp}_{{ist}}$	32,047	0.33	0.47	0	1
$\hbox {RTA}_{{ijt}}$	32,047	0.38	0.49	0	1
$\hbox {Specimens}_{{ist}}$	32,047	0.21	0.41	0	1
$\hbox {Corruption}_{{it}}$	18,305	$-$ 0.59	1.11	$-$ 2.47	1.67

## Footnotes

1 https://www.cites.org/.

2 https://cites.org/eng/resources/ref/suspend.php.

3 Zoonotic diseases can also come from pathogen types other than viruses, e.g. bacteria, prions, fungi and parasites. We focus on viruses as they have relatively few treatment options and are therefore much more of a concern. For the remaining paper content we will use the term “diseases” to mean “viruses” when referring to the Johnson et al. (2020) dataset.

4 The possible trade restrictions, which occur due to an Appendix III listing, is a unilateral decision and can vary in strictness. In our sample, an Appendix III listing and the volume of trade is negatively correlated. However, due to uncertainty surrounding whether or not to interpret species listed in Appendix III as strictly regulated, these 1,604 observations were dropped from our sample. Leaving the Appendix III listing in our sample and assigning it to either the strict Appendix I or less strict Appendix II does not change our findings. Results are available upon request from the authors.

5 The macaca has been found to be easier to breed in captivity than other potential laboratory animals, and in the early 1990’s, a number of macaca breeding companies were established. The industry boomed, by 2008 existing of over 40 companies in China alone, exporting over 170 thousand specimens a year for research use. See https://cites.org/sites/default/files/ndf_material/WG5-CS5.pdf.

6 This has been noticed by CITES, which put the National Legislation Project in place to encourage and assist a country to meet the convention’s minimum requirements to national legislation. See https://cites.org/legislation/National_Legislation_Project for more information on this issue.

7 http://info.worldbank.org/governance/wgi/.
